# Lack of Contribution of p66shc and Its Mitochondrial Translocation to Ischemia-Reperfusion Injury and Cardioprotection by Ischemic Preconditioning

**DOI:** 10.3389/fphys.2017.00733

**Published:** 2017-10-05

**Authors:** Kerstin Boengler, Péter Bencsik, János Palóczi, Krisztina Kiss, Márton Pipicz, Judit Pipis, Péter Ferdinandy, Klaus-Dieter Schlüter, Rainer Schulz

**Affiliations:** ^1^Physiologisches Institut, Justus-Liebig-Universität, Giessen, Germany; ^2^Pharmahungary Group, Szeged, Hungary; ^3^Cardiovascular Research Group, Department of Biochemistry, University of Szeged, Szeged, Hungary; ^4^Department of Pharmacology and Pharmacotherapy, Semmelweis University, Budapest, Hungary

**Keywords:** ischemia/reperfusion, ischemic preconditioning, reactive oxygen species, mitochondria, p66shc

## Abstract

Whereas high amounts of reactive oxygen species (ROS) contribute to cardiac damage following ischemia and reperfusion (IR), low amounts function as trigger molecules in the cardioprotection by ischemic preconditioning (IPC). The mitochondrial translocation and contribution of the hydrogen peroxide-generating protein p66shc in the cardioprotection by IPC is unclear yet. In the present study, we investigated the mitochondrial translocation of p66shc, addressed the impact of p66shc on ROS formation after IR, and characterized the role of p66shc in IR injury *per se* and in the cardioprotection by IPC. The amount of p66shc in subsarcolemmal (SSM) and interfibrillar mitochondria (IFM) isolated from wildtype mouse left ventricles (LV) was determined after 40 min normoxic perfusion and after 30 min ischemia and 10 min reperfusion without and with IPC. The p66shc content in SSM (in % of normoxic controls, *n* = 5) was 174 ± 16% (*n* = 6, *p* < 0.05) after IR, and was reduced to 128 ± 13% after IPC (*n* = 6, *p* = ns). In IFM, the amount of p66shc remained unchanged (IR: 81 ± 7%, *n* = 6; IPC: 110 ± 5%, *n* = 6, *p* = ns). IR induced an increase in ROS formation in SSM and IFM isolated from mouse wildtype LV, which was more pronounced in SSM than in IFM (1.18 ± 0.18 vs. 0.81 ± 0.16, *n* = 6, *p* < 0.05). In mitochondria from p66shc-knockout mice (p66shc-KO), the increase in ROS formation by IR was not different between SSM and IFM (0.90 ± 0.11 vs. 0.73 ± 0.08, *n* = 6, *p* = ns). Infarct size (in % of the left ventricle) was 51.7 ± 2.9% in wildtype and 59.7 ± 3.8% in p66shc-KO hearts *in vitro* and was significantly reduced to 35.8 ± 4.4% (wildtype) and 34.7 ± 5.6% (p66shc-KO) by IPC, respectively. *In vivo*, infarct size was 57.8 ± 2.9% following IR (*n* = 9) and was reduced to 40.3 ± 3.5% by IPC (*n* = 11, *p* < 0.05) in wildtype mice. In p66shc-knockout mice, infarct sizes were similar to those measured in wildtype animals (IR: 56.2 ± 4.3%, *n* = 11; IPC: 42.1 ± 3.9%, *n* = 13, *p* < 0.05). Taken together, the mitochondrial translocation of p66shc following IR and IPC differs between mitochondrial populations. However, similar infarct sizes after IR and preserved infarct size reductions by IPC in p66shc-KO mice suggest that p66shc-derived ROS are not involved in the cardioprotection by IPC nor do they contribute to IR injury *per se*.

## Introduction

An imbalance in the formation and removal of reactive oxygen species (ROS) leads to oxidative stress, which plays a role in the development of cardiovascular diseases, such as hypertension (Chen et al., 2017), hypertrophy (Dai et al., 2011; Sag et al., 2014), heart failure (Akhmedov A. T. et al., 2015), and myocardial injury following ischemia and reperfusion (IR) (Granger and Kvietys, 2015). During IR, a certain amount of ROS is generated during ischemia, whereas the majority of ROS is formed at the onset of reperfusion (Zweier et al., 1987; Bolli et al., 1989). High amounts of ROS contribute to myocardial injury and ultimately cell death via detrimental effects on proteins and lipids and also on the histone-free mitochondrial DNA. However, ROS do not only participate in myocardial damage, they also function as trigger molecules in the cardioprotection by ischemic preconditioning (IPC). Here, a modest ROS formation is suggested to activate signal transduction cascades which finally confer protection against the burst of ROS at reperfusion. Indeed, ROS scavenging during the preconditioning cycles of IR as well as prior to reperfusion abolish the infarct size reduction by IPC (Skyschally et al., 2003; Liu et al., 2008). It is generally accepted that mitochondria represent the predominant source of ROS. Within mitochondria, ROS are formed by the electron transport chain (ETC)—especially from ETC complexes I, II and III (Barja, 1999)—with around 0.2% of the oxygen consumed by the ETC used for ROS formation (St-Pierre et al., 2002). In addition to the ETC, mitochondrial ROS are also produced by monoamino oxidases (MAO), which transfer electrons from amine compounds to oxygen and thereby generate hydrogen peroxide.

Another protein contributing to mitochondrial ROS formation is p66shc, an ubiquitously expressed member of the spontaneous human combustion (shc) family. Together with p46shc and p52shc, p66shc represents an isoform encoded by the human shcA locus. The structure of p66shc includes an aminoterminal CH2 domain (collagen homology domain), followed by a phosphotyrosine binding (PTB) domain, another collagen-homology (CH1) domain, and a carboxyterminal src-homology (SH2) domain. The PTB domain allows the interaction with tyrosine-containing peptides, the CH1 domain of p66shc contains two major tyrosine phosphorylation sites, whereas the SH2 domain is important for protein-protein interactions. The important phosphorylation site serine 36 is located in the CH2 domain of p66shc. Under basal conditions, the majority of p66shc resides in the cytosol, but translocates into the mitochondria upon stress signals (Pinton et al., 2007). For this translocation, the phosphorylation of p66shc at serine 36 by protein kinase C beta (PKCβ) is important (Pinton et al., 2007). Within mitochondria, p66shc is present in the intermembrane space. Here, p66shc oxidizes reduced cytochrome c and thereby catalyzes the reduction of oxygen to hydrogen peroxide (Giorgio et al., 2005). Accordingly, p66shc-deficient cells have decreased levels of ROS (Trinei et al., 2002; Carpi et al., 2009). The reduced ROS formation in p66shc-deficient mice has been suggested to prolong the life span of these animals (Migliaccio et al., 1999), however, when the mice are housed under more natural conditions this effect is abolished (Giorgio et al., 2012). p66shc-mediated ROS formation is linked to cardiovascular pathologies such as hypertrophy (Graiani et al., 2005) and heart failure (Rota et al., 2006) (for review see Di Lisa et al., 2017). Also, heart-rupture is reduced in p66shc-deficient mice following myocardial infarction (Baysa et al., 2015). The measurement of myocardial damage following IR in wildtype and p66shc-knockout mice shows conflicting results: whereas in one study the ablation of p66shc elicits cardiac protection (Carpi et al., 2009), another study displays larger infarcts in p66shc-deficient mice following IR (Akhmedov A. et al., 2015). Studies on the role of p66shc in the cardioprotection by IPC *in vivo* are still lacking.

In the present study, we investigated the translocation of the protein into mitochondrial subpopulations after IR and IPC. Also, the p66shc-mediated ROS formation induced by IR was studied. In addition, we characterized the impact of p66shc on the cardioprotection by IPC in mouse hearts *in vitro* and *in vivo*.

## Materials and methods

### Animals

The present study conforms to the Guide for the Care and Use of Laboratory Animals published by the US National Institutes of Health (NIH publication No. 85–23, revised 1996) and was approved by the animal welfare office of the Justus-Liebig-University Giessen as well as the National Scientific Ethical Committee on Animal Experimentation, Budapest, Hungary. In the study, 12–22 weeks old male and female C57Bl6/J mice (25–30 g, Janvier, Le Genest-Saint-Isles, France) and p66shc knockout (p66shc-KO) mice were used. Mice were kept in dark/light cycles of 12 h each and had free access to standard chow and drinking water.

### Ischemia/reperfusion *in vitro*

Mice were anesthetized with 5% isoflurane and killed by cervical dislocation. Thereafter, hearts were rapidly excised and the aorta was cannulated for retrograde perfusion with an Aortic Cannula for mouse hearts (Ø 1 mm, Hugo Sachs Elektronik-Harvard Apparatus, March, Germany) connected to a Langendorff perfusion system. Hearts were perfused with 37°C warm modified Krebs Henseleit buffer (containing in mM: NaCl 118, KCl 4.7, MgSO_4_ 0.8, KH_2_PO_4_ 1.2, glucose 5, CaCl_2_ 2.5, NaHCO_3_ 25, pyruvate 1.9, continuously gased with 95% O_2_, 5% CO_2_, pH 7.4) at a constant perfusion pressure of 70 mmHg (transduced by a Replacement Transducer Head for APT300 Pressure Transducer, Hugo Sachs Elektronik-Harvard Apparatus). A balloon was inserted into the left ventricle and was connected to a pressure transducer (Combitrans 1-fach Set Mod.II University Giessen, B. Braun, Melsungen, Germany) for assessment of ventricular performance. The balloon was inflated to yield a left ventricular end-diastolic pressure of 12–14 mmHg, which was kept constant thereafter. Hearts were paced during measurements at 600 bpm. Left ventricular developed pressure (LVDP, systolic pressure—diastolic pressure) was recorded. Perfused hearts were left to stabilize for 5 min. Ischemia was induced by stopping flow and pacing. The following protocols were performed:

a) p66shc translocation and ROS formation

Normoxia: 40 min normoxia

IR: 30 min ischemia, 10 min reperfusion

IPC: Three times 3 min ischemia, 5 min reperfusion, followed by 30 min ischemia and 10 min reperfusion

At the end of the protocol, hearts were used to isolate mitochondria

b) Infarct size determination

IR: 45 min ischemia, 120 min reperfusion

IPC: Three times 3 min ischemia, 5 min reperfusion, followed by 45 min ischemia and 120 min reperfusion

After 120 min of reperfusion, the hearts were removed from the perfusion apparatus and frozen at −20°C for 30 min. Subsequently, hearts were cut in 7–8 slices and incubated in 1.2% triphenyl-tetrazolium chloride for 20 min at 37°C. Heart slices were then fixated in 7% formalin at room temperature overnight. Digital images were taken from both sides of the heart slices with a M60 microscope (Leica, Wetzlar, Germany) at 2.5-fold magnification. Infarct size was determined by planimetrie using the Leica Application Suite LAS version 4.6 (Leica).

The use of either 30 or 45 min ischemia was due to the necessity to compare data of p66shc translocation with previous studies (were 30 min ischemia were analyzed, Yang et al., 2014) and to induce substantial myocardial infarction in order to demonstrate effective cardioprotection by IPC (45 min ischemia).

### Ischemia/reperfusion *in vivo*

Mice were weighed (weight range 22.1 ± 1.0–24.7 ± 1.1 g, *p* = ns between groups) and anesthetized with sodium pentobarbital (Euthasol, Produlab Pharma b.v., Raamsdonksveer, The Netherlands; 90 mg/kg bolus dose followed by 15–20 mg/kg when required during the experiment). The hair in the neck and chest area was removed by using a depilatory cream. Maintenance of body core temperature was assisted using a constant temperature heating pad. The trachea was intubated with a plastic cannula connected to a rodent ventilator (Model Minivent 845, Harvard Apparatus, Holliston, MA). The animals were ventilated with room air, volume and rate set-ups accorded to the recommendation of the manufacturer (100–240 μL, 120–150 breath/min according to the weight of the animal). Surface-lead ECG and body core temperature were monitored throughout the experiments to ensure the stability of the preparation (Haemosys data acquisition system, Experimetria, Budapest, Hungary). The heart rates ranged from 429 ± 17 to 451 ± 20 bpm and were not significantly different between groups. The chest was opened at the 4th intercostal space and an 8-0 Prolene suture was placed around the middle portion of the left anterior descending branch (LAD) of the left coronary artery. Then the suture was looped and a piece of PE-10 cannula was placed into the loop. For coronary artery occlusion and reperfusion, both strands of the suture were pulled and fixed thereby pressing the plastic cannula onto the surface of the heart directly above the coronary artery, and then released. Mice were subjected to 45 min occlusion of the left coronary artery (test ischemia) and then released to develop acute myocardial infarction. In IPC groups, mice were subjected to 5 min ischemia/5 min reperfusion in four cycles prior to test ischemia. To ensure recanalization of the occluded vessel, sodium heparin was administered i.p. at 100 U/kg dose three times during the surgeries: 45 min before test ischemia; 5 min before the onset of reperfusion, and at the 115th min of reperfusion.

After 120 min of reperfusion, risk area was re-occluded, and mice were injected with 0.4 ml of 2% Evans blue dye through the apex of the left ventricle. Following Evans staining, hearts were isolated, right ventricle was removed and left ventricles (LV) were cut into seven transversal slices. Heart slices were washed in PBS buffer for 1 min to remove excess dye and then incubated in 1% triphenyl-tetrazolium-chloride for 10 min at 37°C followed by formalin fixation for 10 min. Digital images were taken from both surface of heart slices by a Nikon DSLR camera (Nikon Corporation, Tokyo, Japan). Planimetric evaluation was carried out to determine infarct size using InfarctSize™ software version 2.5, (Pharmahungary, Szeged, Hungary).

### Isolation of mitochondria

Subsarcolemmal (SSM) and interfibrillar mitochondria (IFM) were isolated as previously described (Boengler et al., 2009). All steps were performed at 4°C. Hearts were washed in buffer A (100 mM KCl, 50 mM 3-[N-Morpholino]-propanesulfonic acid (MOPS), 5 mM MgSO4, 1 mM ATP, 1 mM EGTA, pH 7.4), weighed, the tissue was minced in 10 ml/g buffer A with scissors and was then disrupted with a Potter-Elvejhem tissue homogenizer. The homogenate was centrifuged for 10 min at 800 g. The resulting supernatant, which contained the SSM, was centrifuged for 10 min at 8,000 g. The sedimented mitochondria were washed in buffer A and were resuspended in a small volume of buffer A. The sediment of the first centrifugation, which contained the IFM, was resuspended in buffer A (10 ml/g tissue). The protease nagarse was added (Bacterial type XXIV, Sigma, 8 U/g), incubated at 4°C for 1 min and the samples were then disrupted using a Potter-Elvejhem tissue homogenizer. Subsequently, samples were centrifuged for 10 min at 800 g, and IFM were collected by centrifugation of the supernatant for 10 min at 8,000 g. The sedimented IFM were washed by resuspension in buffer A and centrifugation (8,000 g for 10 min), and were finally resuspended in buffer A. These mitochondrial preparations were used to study ROS formation. To analyse the amount of p66shc in SSM and IFM by Western blot, mitochondria were further purified by layering them on top of a 30% Percoll solution in isolation buffer (in mM: sucrose 250; HEPES 10; EGTA 1; pH 7.4) and subsequent ultracentrifugation at 35,000 g for 30 min at 4°C. The mitochondrial band was collected, washed twice in isolation buffer by centrifugation at 8,000 g for 5 min, and the purified mitochondria were stored at −80°C.

### ROS formation

ROS formation was measured as described previously (Boengler et al., 2017). Fifty microgram mitochondria (SSM and IFM) isolated after normoxia or IR were transferred to incubation buffer supplemented with 5 mM glutamate and 2.5 mM malate, 50 μM Amplex UltraRed (Invitrogen, Eugene, OR), and 0.1 U/ml horseradish peroxidase. The fluorescence was measured continuously for 4 min with a Cary Eclipse spectrophotometer (Agilent Technologies, Santa Clara, CA) at the excitation/emission wavelengths of 565/581 nm, respectively. As positive control served control mitochondria supplemented with 2 μM of the complex I inhibitor rotenone. Background fluorescence of the buffer without mitochondria was subtracted and the slope fluorescence in arbitrary units/time (4 min) was calculated.

### Western blot analysis

Isolated SSM, IFM, or left ventricular tissue sections were lysed in 1 × Cell Lysis buffer (25 mM Tris, 150 mM NaCl, 1 mM EDTA, 1% NP-40, 5% glycerol, pH 7.4) supplemented with 1X PhosStop and Complete inhibitors (Roche, Basel, Switzerland) as well as 1 μM neocuproine. Protein concentration was determined using the Lowry assay. Thirty microgram proteins were electrophoretically separated on 10% Bis/Tris gels and proteins were transferred to nitrocellulose membranes. After blocking, membranes were incubated with rabbit polyclonal anti-human/rat SHC antibodies (BD Biosciences), rabbit polyclonal anti-human voltage dependent anion channel (VDAC, Acris, Rockville MD), or rabbit polyclonal anti-human manganese superoxide dismutase antibodies (MnSOD, Merck Millipore, Darmstadt, Germany). After washing and incubation with the respective secondary antibodies, immunoreactive signals were detected by chemiluminescence (SuperSignal West Femto or SuperSignal West Pico Chemiluminescent Substrate, ThermoFisher) and quantified using Scion Image software (Frederick, MD). The purity of the mitochondrial preparations was determined as the absence of immunoreactivity for Na^+^/K^+^-ATPase (sarcolemma), sarcoplasmic/endoplasmic reticulum calcium ATPase (sarcoplasmic reticulum), histone deacetylase 2 (nucleus), and glycerinaldehyde-3-phosphate dehydrogenase (cytosol), data not shown.

### Statistics

Data are shown as mean ± SEM and a *p* < 0.05 is considered to indicate a significant difference. Data on the mitochondrial content of p66shc in SSM and IFM (basal, following IR and IPC) were compared by non-parametric Rank Sum test. Data on ROS formation, EDP, LVDP, the recovery of LVDP, area at risk *in vivo*, as well as on infarct size determination *in vitro* and *in vivo* were analyzed by two-way ANOVA, following Bonferroni corrections. The program SigmaStat 3.5 (Systat, Software GmbH, Erkrath, Germany) was used for statistical analysis.

## Results

**Figure 1:**
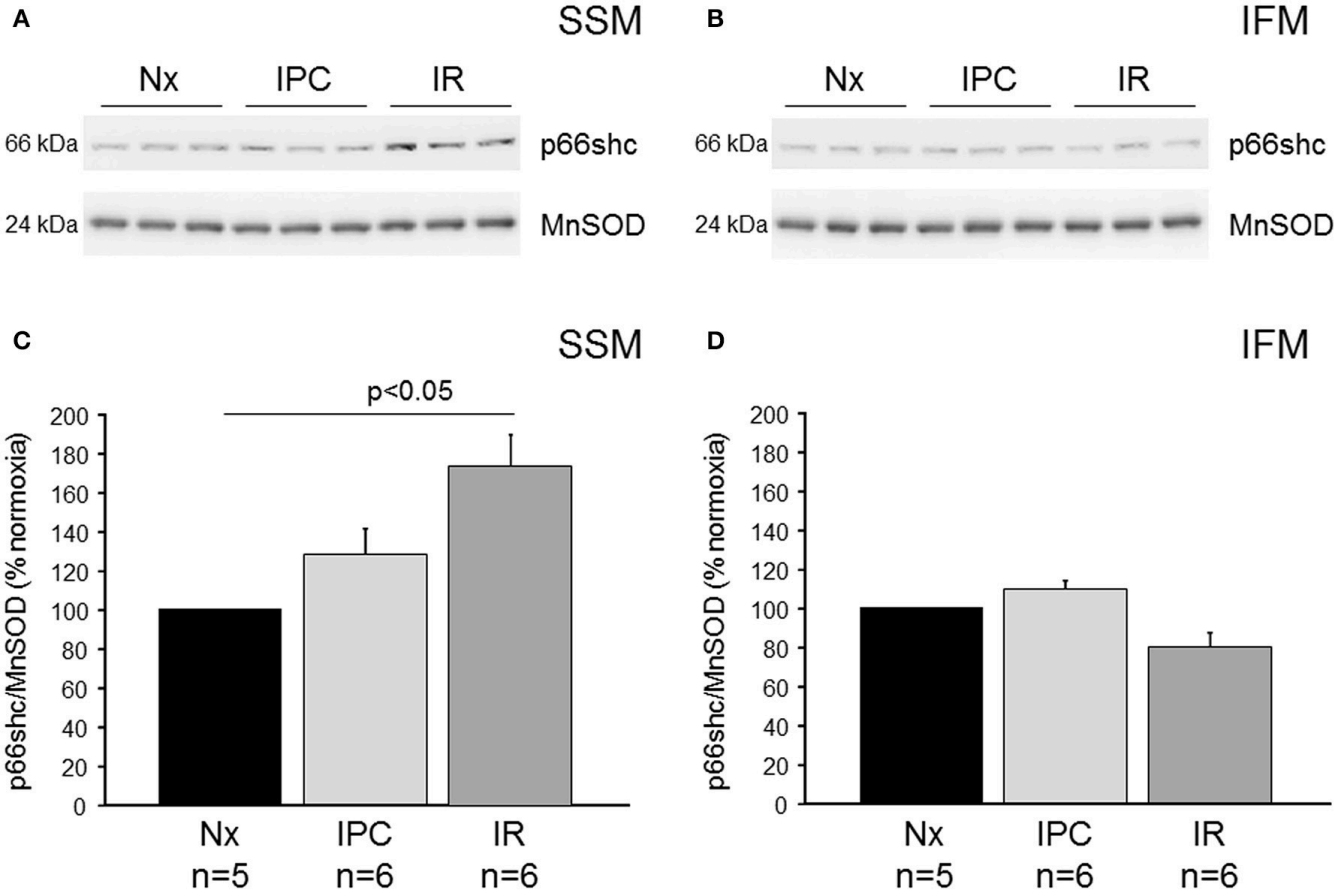
Mitochondrial p66shc translocation following ischemia/reperfusion or ischemic preconditioning. Western blot analysis was performed for p66shc and the mitochondrial marker protein MnSOD (manganese superoxide dismutase) on SSM **(A)** and IFM **(B)** isolated from wildtype mice undergoing normoxia (Nx), ischemia/reperfusion (IR) or IR with ischemic preconditioning (IPC). Bar graphs represent the ratios of p66shc over MnSOD in SSM **(C)** and IFM **(D)** isolated after Nx, IR, or IPC.

To study the mitochondrial translocation of p66shc, isolated mouse hearts were perfused under normoxic conditions or subjected to IR (30 min ischemia, 10 min reperfusion) without and with IPC. SSM and IFM were isolated and analyzed for their p66shc content by Western blot (Figure 1). In SSM, IR induced an increased translocation of p66shc into the mitochondria, however, following IPC the p66shc content was reduced to that of normoxic controls. In contrast to SSM, the amount of p66shc in IFM was not affected by IR or IPC.

**Figure 2:**
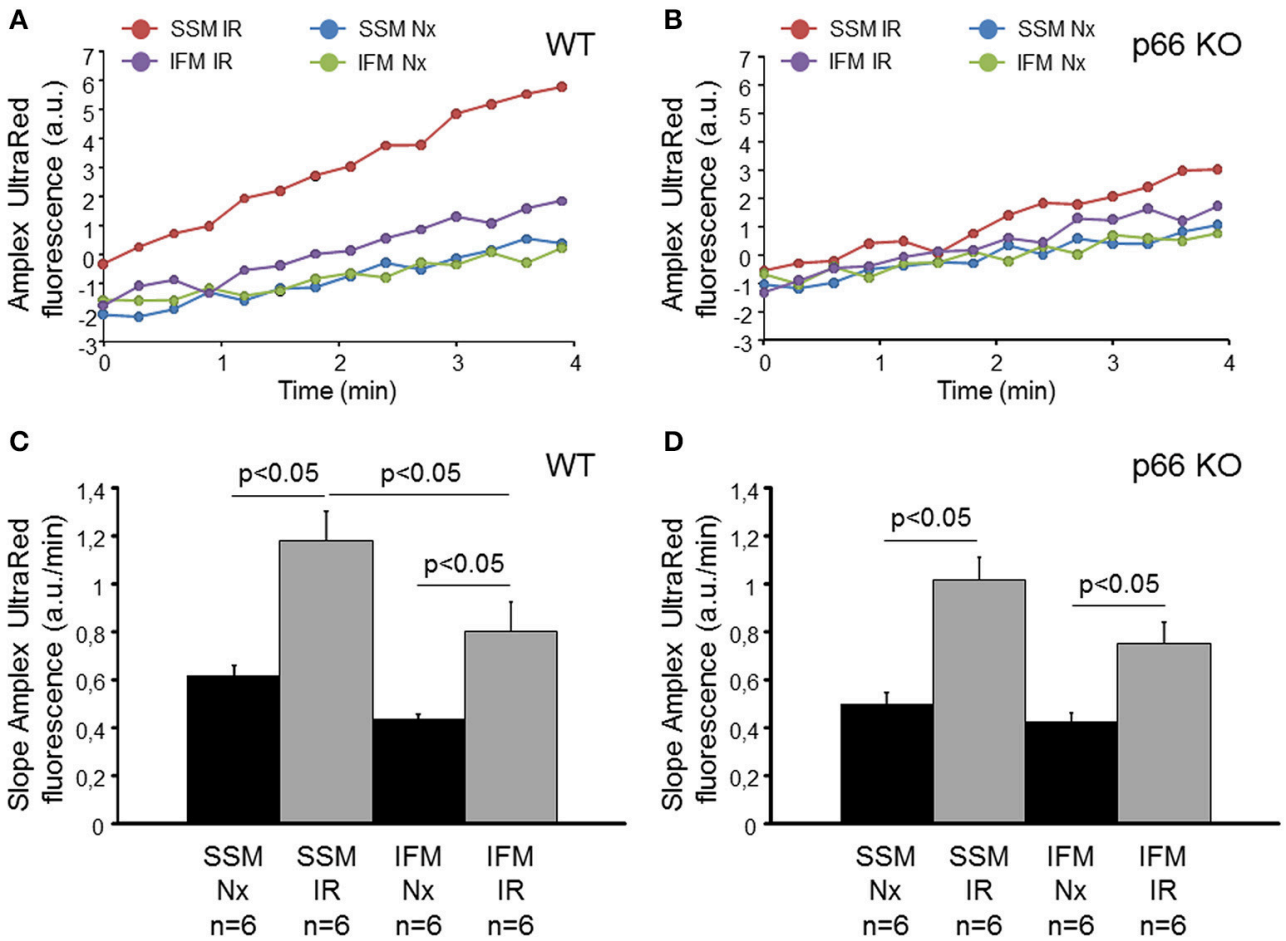
ROS formation in SSM and IFM isolated from mouse hearts following normoxia or ischemia/reperfusion *in vitro*. Original traces showing Amplex UltraRed fluorescence in SSM and IFM isolated after normoxia (Nx) or ischemia/reperfusion (IR) in wildtype (WT, **A**) or p66shc knockout (p66 KO, **B**) hearts *in vitro*. Bar graphs represent the slope of the Amplex UltraRed fluorescence measured for 4 min in WT **(C)** and p66 KO **(D)** mitochondria.

To investigate whether or not the mitochondrial amount of p66shc correlates with the ROS formation following IR, isolated hearts from wildtype (WT) or p66shc-KO mice underwent normoxia or IR. Subsequently, SSM and IFM were isolated and ROS formation was measured as the increase in the Amplex UltraRed fluorescence (Figure 2). Under normoxic conditions, ROS formation tended to be higher in SSM compared to IFM isolated from both WT and p66shc-KO hearts without reaching statistical significance. Following IR, ROS formation increased in both SSM and IFM from WT and p66shc-KO hearts, however, the raise in ROS formation in SSM compared to IFM was more pronounced in WT than in p66shc-KO mitochondria. When ROS formation was stimulated by the addition of rotenone, there were no differences in the slope of the Amplex UltraRed fluorescence (in arbitrary units/min) between SSM and IFM isolated from WT (SSM Nx: 1.6 ± 0.2; SSM IR: 1.8 ± 0.2; IFM Nx: 2.3 ± 0.5; IFM IR: 1.9 ± 0.3, *n* = 6, *p* = ns) and p66shc-KO hearts (SSM Nx: 2.16 ± 0.3; SSM IR: 2.2 ± 0.2; IFM Nx: 1.9 ± 0.3; IFM IR: 2.5 ± 0.3, *n* = 6, *p* = ns).

**Figure 3:**
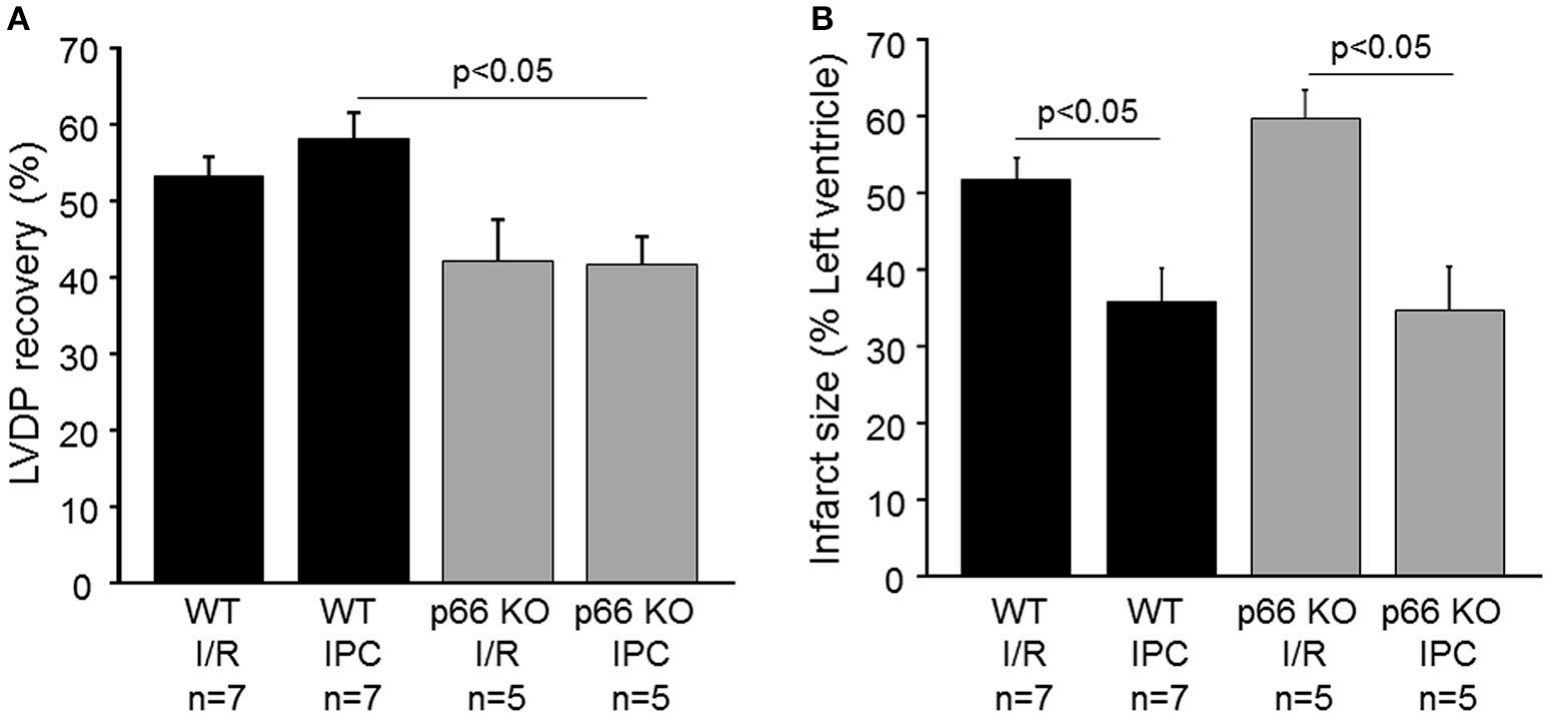
Influence of p66shc on myocardial function, IR injury and cardioprotection *in vitro*. **(A)** Left ventricular developed pressure (LVDP) at the end of reperfusion in % of that at the end of the stabilization period in wildtype (WT) and p66shc knockout (p66 KO) mice undergoing ischemia/reperfusion (IR) or ischemic preconditioning (IPC). **(B)** Infarct size (in % of left ventricle) in WT and p66shc-KO mice subjected to IR or IPC.

The impact of p66shc on left ventricular function was determined in isolated WT and p66shc-KO hearts subjected to IR without or with IPC. Under baseline conditions (i.e., at the end of the stabilization period), end-diastolic pressure and LVDP were not different between groups (Table 1). The recovery of the LVDP at the end of reperfusion was more pronounced in WT hearts undergoing IPC than in p66shc-KO hearts (Figure 3A, Table 1). However, the improved functional recovery was not a consequence of altered infarct size, since IPC induced a similar infarct size reduction in WT and in p66shc-KO hearts *in vitro* (Figure 3B). Myocardial infarction after IR alone was not different between WT and p66shc-KO hearts.

**Table 1:** Summary of the baseline parameters and hemodynamic data throughout ischaemia-reperfusion protocols *in vitro*.

**Genotype**	**Protocol**	***n*-value**	**Body weight (g)**	**Heart weight/body weight (mg/g)**	**EDP (mm Hg)**			**LVDP (mm Hg)**		
					**basal**	**10 min reperfusion**	**End of reperfusion**	**basal**	**10 min reperfusion**	**End of reperfusion**
WT	IR *in vitro*	7	28.9 ± 1.2	6.25 ± 0.27	12.8 ± 0.4	51.4 ± 6.1	26.3 ± 4.1	107.0 ± 3.7	59.8 ± 11.9	56.7 ± 2.8
WT	IPC *in vitro*	7	27.7 ± 1.5	6.51 ± 0.24	11.1 ± 0.7	25.7 ± 2.5^*^	14.2 ± 0.9^*^	101.8 ± 7.7	67.5 ± 4.2	59.5 ± 6.4
p66 KO	IR *in vitro*	5	25.2 ± 0.5	6.91 ± 0.51	12.0 ± 1.0	62.7 ± 11.3	31.0 ± 4.4	90.6 ± 10.5	32.7 ± 8.3^*^	36.0 ± 2.7^*^
p66 KO	IPC *in vitro*	5	26.0 ± 0.9	6.30 ± 0.48	11.6 ± 0.9	33.8 ± 16.0	19.0 ± 7.4	95.4 ± 10.3	31.0 ± 8.1^**^	38.7 ± 3.5^**^

*Enddiastolic pressure (EDP) and left ventricular developed pressure (LVDP) in wildtype and p66shc knockout (p66 KO) hearts undergoing IR without and with ischemic preconditioning (IPC). Basal data were collected at the end of the stabilization period*.

* *p < 0.05 vs. I/R WT*,

** *p < 0.05 vs. IPC WT*.

**Figure 4:**
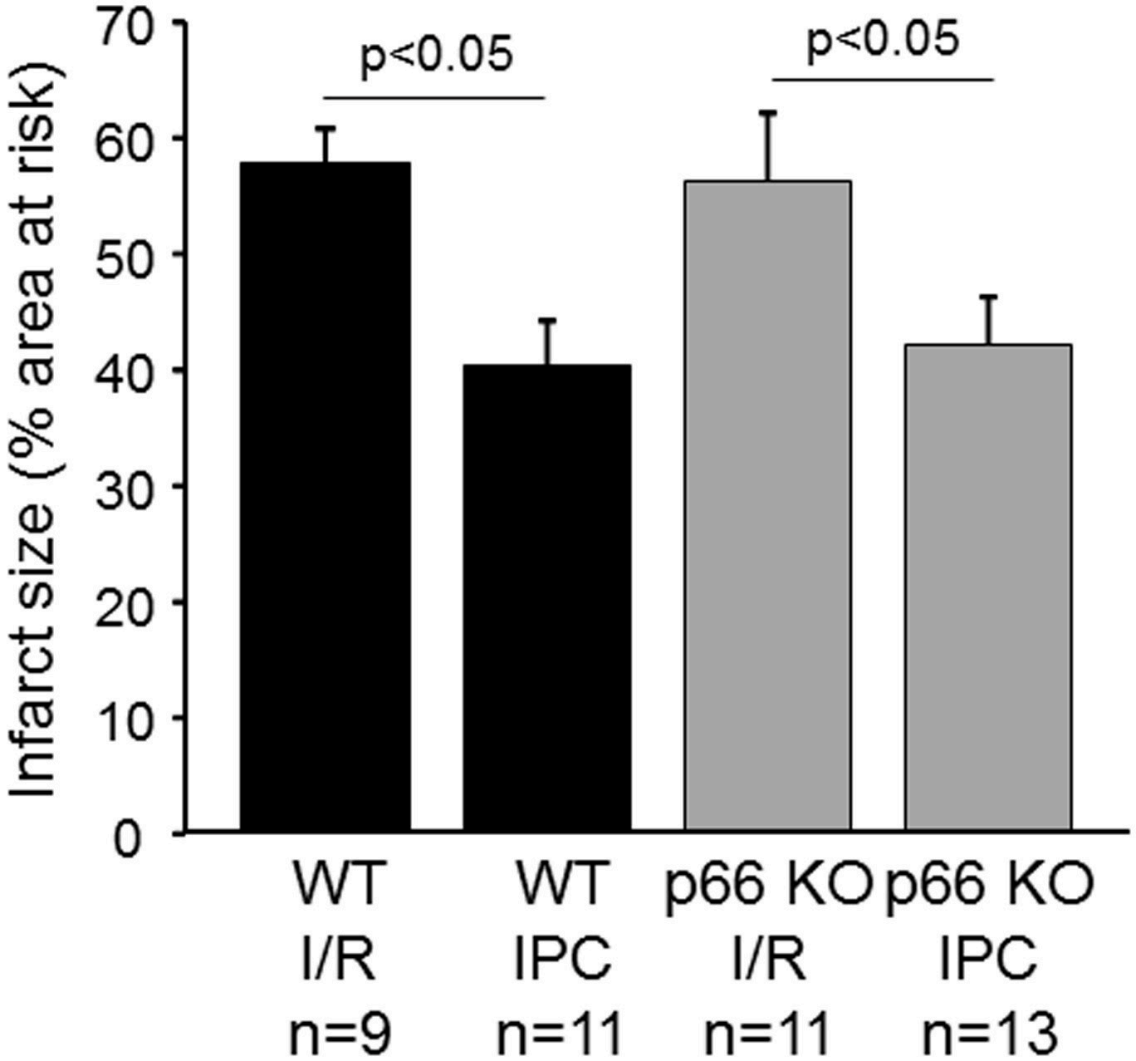
Influence of p66shc on myocardial IR injury and cardioprotection by ischemic preconditioning *in vivo*. Infarct size (in % of the area at risk) in WT and p66shc knockout (p66 KO) mice subjected to ischemia/reperfusion (IR) or ischemic preconditioning (IPC).

To study the role of p66shc in the cardioprotection by IPC *in vivo*, the LAD branch of the left coronary artery was reversibly occluded in WT and p66shc-KO mice to induce IR without and with IPC. The area at risk (in % of the left ventricle) was not different between groups (WT, IR: 23.2 ± 2.4, *n* = 9; WT IPC: 34.5 ± 5.2, *n* = 11; p66shc-KO IR: 26.9 ± 2.5, *n* = 11; p66shc-KO IPC: 27.9 ± 2.7, *n* = 13, *p* = ns). Also, there was no significant difference in infarct size after IR between WT and p66shc-KO mice (Figure 4). However, with IPC infarct size was significantly reduced in both WT and p66shc-KO mice demonstrating effective cardioprotection not only in WT but also in p66shc-KO mice *in vivo* (Figure 4).

## Discussion

The present study demonstrates that the translocation of p66shc after IR or IPC differs between mitochondrial subpopulations. An increase in the mitochondrial level of p66shc in SSM is associated with enhanced ROS formation after IR. However, the altered mitochondrial amounts of p66shc after IR or IPC had no consequences for infarct development *per se* or the cardioprotection, since p66shc knockout hearts showed an effective infarct size reduction by IPC both *in vitro* and *in vivo*.

The presence of p66shc has been described in mitochondria of several cell types, including mouse embryonic fibroblasts (Nemoto et al., 2006), human endothelial cells (Paneni et al., 2015; Spescha et al., 2015; Zhu et al., 2015), and mitochondria isolated from cardiac tissue (Yang et al., 2014). Cardiomyocytes contain at least two mitochondrial subpopulations, the SSM and IFM, which differ in form and function (Palmer et al., 1977, 1986; Boengler et al., 2009). When analyzing the presence of p66shc in mitochondria of ventricular origin, only SSM have been studied so far (Yang et al., 2014). In the present study, we detected p66shc not only in cardiac SSM but also in IFM. Under basal conditions, the majority of p66shc resides in the cytosol and a translocation of the protein into the mitochondrial intermembrane space occurs under stress conditions, among them IR (Giorgio et al., 2005; Zhu et al., 2015). A previous study demonstrates that the translocation of p66shc into SSM is dependent on the duration of IR in guinea pig hearts (Yang et al., 2014). Here, 30 min of ischemia were not sufficient to increase the mitochondrial amount of p66shc, whereas 30 min ischemia and 10 min reperfusion enhanced the mitochondrial content of the protein. In the present study, the increased mitochondrial amount of p66shc after 30 min ischemia and 10 min reperfusion in SSM was confirmed, but this translocation was specific for SSM since the mitochondrial amounts of p66shc in IFM was not affected by IR.

The import of p66shc into mitochondria requires the phosphorylation at serine 36 by protein kinase C beta (PKCβ), and the subsequent prolyl-isomerization by peptidyl-prolyl cis-trans isomerase 1 (Pin1) is important. Indeed, it has already been shown that 30 min IR induces the activation/phosphorylation of PKCβ and simultaneously that of p66shc at serine 36, and that the inhibition of PKCβ decreases p66shc phosphorylation and the mitochondrial translocation of the protein (Kong et al., 2008; Yang et al., 2014). However, serine 36 phosphorylation of p66shc may also require c-Jun terminal kinase activity (Khalid et al., 2016). In human umbilical vein endothelial cells, hypoxia/reoxygenation is associated with increased phosphorylation and mitochondrial translocation of p66shc (Zhu et al., 2015). Here, the increased p66shc phosphorylation is attributed to decreased activity of phosphatase 2A rather than to increased activity of PKCβ. The mitochondrial translocation of p66shc after intestinal IR injury is abrogated following the inhibition of Pin1 leading to improved survival (Feng et al., 2017). Under high glucose conditions, the phosphorylation and mitochondrial translocation of p66shc is facilitated by a Sirtuin 1-regulated lysine acetylation (Kumar et al., 2017). Although we tried to measure serine 36 phosphorylation of p66shc by Western blot and immunoprecipitation in the present study, but were unable to detect specific signals with available antibodies (data not shown), we cannot correlate p66shc phosphorylation with the mitochondrial amount of the protein.

The ablation of p66shc is associated with a reduced ROS formation after IR in the brain (Spescha et al., 2013) as well as in the heart (Carpi et al., 2009). However, one study also shows that the deletion of p66shc (via siRNA or by genetic ablation) has no influence on myocardial ROS formation following IR (Spescha et al., 2015). In our study, we found an increase in ROS formation after IR compared to normoxia in SSM and IFM of wildtype and p66shc-deficient mice. In wildtype mice, this increase was more pronounced in SSM than in IFM and therefore correlated with the mitochondrial translocation of p66shc. However, in mitochondria isolated from p66shc-deficient mice ROS formation was not different in SSM and IFM after IR indicating that p66shc contributes sufficient amounts to the ROS formation induced by myocardial IR.

Since ROS are known to contribute to either myocardial damage or protection—depending on their timing and their amount—p66shc represents an interesting target to be studied in IR and protection from it. p66shc induces opening of the mitochondrial permeability transition pore, which leads to swelling of the organelle, rupture of the outer mitochondrial membrane and finally cell death (Giorgio et al., 2005). Therefore, the deletion of p66shc has been suggested to be protective in IR injury, and indeed IR in the brain induced by transient middle cerebral artery occlusion results in reduced stroke size in p66shc-KO mice or in WT mice after post-ischemic silencing of p66shc compared to that in control mice (Spescha et al., 2013, 2015). Also, muscle fiber necrosis is reduced in p66shc-deficient mice after hindlimb IR (Zaccagnini et al., 2004). In the heart, the data on the role of p66shc in IR injury are controversial. Whereas, one study demonstrates the maintenance of cell viability and reduced oxidative stress in p66shc-deficient hearts following IR *in vitro* (Carpi et al., 2009), the measurement of myocardial infarction in p66shc-deficient mice *in vivo* shows larger infarct sizes after IR compared to that in wildtype mice (Akhmedov A. et al., 2015). However, myocardial infarction is untypically small in this study, and the increase in myocardial damage is only evident after short term ischemia (30 min), whereas with the prolongation of ischemia to 45 or 60 min no differences in infarct sizes occur between wildtype and p66shc-deficient mice. In the present study, we determined the infarct sizes of wildtype and p66shc-deficient mice undergoing IR (with 45 min of ischemia) *in vitro* and *in vivo* and we observed similar myocardial infarction in both genotypes indicating that p66shc—and the p66shc-induced ROS formation—does not contribute to IR injury *per se*.

Due to the important role of ROS in IR injury and in the protection by IPC, p66shc represents a putative target of such protective intervention. Indeed, in cortical cells chemical preconditioning induces serine 36 phosphorylation of p66shc, subsequent mitochondrial translocation of the protein and finally reduces cell death (Brown et al., 2010). Whereas, this study suggests a protective role of p66shc in preconditioning, another study demonstrates that IPC in the liver is protective against IR injury via a pathway involving the Sirtuin 1-mediated downregulation of p66shc (Yan et al., 2014). In the present study, we measured the translocation of p66shc into mitochondria after perfusion of isolated wildtype hearts under normoxic control conditions, after IR and as well as after IPC and found that whereas IR and IPC did not alter the mitochondrial amount of p66shc in IFM, the IR-induced increase of p66shc in SSM was abrogated after IPC. Thus, the inhibition of mitochondrial p66shc import by IPC may reduce myocardial ROS formation to such amounts which are necessary for triggering cardioprotection.

In addition, the present study addressed the influence of p66shc on myocardial function and the infarct size development following IR without and with IPC *in vitro* and *in vivo*.

Whereas the recovery of the LVDP was improved in wildtype compared to p66shc-deficient mice after IPC, the enhanced functional recovery was not a consequence of altered myocardial infarction, since IPC reduced infarct sizes to similar extents in both genotypes *in vitro*. Comparable results were obtained in the *in vivo* situation where IPC was equally cardioprotective in wildtype and in p66shc-deficient mice. Therefore, despite the putative normalization of the IR-induced increase of ROS by IPC in SSM, p66shc-mediated ROS formation is no prerequisite for the cardioprotection by IPC. The role of p66shc in IPC in the heart has previously been investigated in one study only (Carpi et al., 2009). Here, myocardial damage was assessed as the release of lactate dehydrogenase (LDH) from isolated hearts *in vitro*. Compared to wildtype mice, LDH release was already reduced in p66shc-deficient mice after IR and was not further affected by IPC. Therefore, it is difficult to assess whether or not IPC was capable to additionally decrease LDH release.

Our data demonstrate that in healthy hearts p66shc is of no importance for myocardial I/R injury and that the protein is also not involved in the cardioprotection by classical ischemic preconditioning. However, alterations in p66shc expression/phosphorylation occur in pathological conditions in humans, such as in muscular pericytes of diabetic patients (Vono et al., 2016), in peripheral blood monocytes and renal tissue biopsies of patients with diabetic nephropathy (Xu et al., 2016), and also in peripheral blood monocytes of patients with acute coronary syndrome, but not with stable coronary artery disease (Franzeck et al., 2012). Since such risk factors and co-morbidities may abrogate the cardioprotection by preconditioning (Ferdinandy et al., 2014), it remains to be elucidated whether p66shc contributes toward cardioprotection under pathological conditions.

Taken together, our study demonstrates that within cardiac mitochondria p66shc is present in SSM as well as in IFM. The IR-induced translocation of p66shc into SSM correlates with the ROS formation in this mitochondrial subpopulation. However, ROS generation by p66shc is not important for myocardial injury, since the ablation of p66shc does not influence infarct size after IR *per se*. Whereas, IPC normalizes the IR-induced increase of p66shc in SSM, this process has no relevance for cardioprotection since p66shc-deficient mice show effective infarct size reduction *in vitro* and *in vivo*.

## Author contributions

KB designed and performed the research on isolated mitochondria; PB, JaP, KK, MP, and JuP performed the research on myocardial infarction *in vivo*; PF, KS, and RS designed and supervised the research. All authors analyzed the data, drafted the manuscript, and approved the final version of the manuscript.

### Conflict of interest statement

RS received research grants from Zealand Pharma and honoraria for lectures and advisory boards from AstraZeneca, Recordati, Sanofi, and Servier. PF is a founder and CEO of Pharmahungary Group. The other authors declare that the research was conducted in the absence of any commercial or financial relationships that could be construed as a potential conflict of interest.
